# Accuracy Validation of a Sensor-Based Inertial Measurement Unit and Motion Capture System for Assessment of Lower Limb Muscle Strength in Older Adults—A Novel and Convenient Measurement Approach

**DOI:** 10.3390/s24186040

**Published:** 2024-09-18

**Authors:** Ye Zhu, Haojie Li, Xie Wu, Nan Chen

**Affiliations:** 1 School of Exercise and Health, Shanghai University of Sport, Shanghai 200438, China; zhuye0425@163.com (Y.Z.);; 2 Chongming Hospital Affiliated to Shanghai University of Medicine and Health Sciences, Shanghai 202150, China

**Keywords:** inertial capture sensors, elderly muscle strength, novel measurement modalities, quality of life, sit-to-stand transfer, infrared motion capture, joint angles, joint moments

## Abstract

(1) Background: The aim of this study was to assess lower limb muscle strength in older adults during the transfer from sitting to standing (STS) using an inertial measurement unit (IMU). Muscle weakness in this population can severely impact function and independence in daily living and increase the risk of falls. By using an IMU, we quantified lower limb joint moments in the STS test to support health management and individualized rehabilitation program development for older adults. (2) Methods: This study involved 28 healthy older adults (13 males and 15 females) aged 60–70 years. The lower limb joint angles and moments estimated using the IMU were compared with a motion capture system (Mocap) (pair *t*-test, ICC, Spearman correlations, Bland–Altman plots) to verify the accuracy of the IMU in estimating lower limb muscle strength in the elderly. (3) Results: There was no significant difference in the lower limb joint angles and moments calculated by the two systems. Joint angles and moments were not significantly different (p > 0.05), and the accuracy and consistency of the IMU system was comparable to that of the Mocap system. For the hip, knee, and ankle joints, the ICCs for joint angles were 0.990, 0.989, and 0.885, and the ICCs for joint moments were 0.94, 0.92, and 0.89, respectively. In addition, the results of the two systems were highly correlated with each other: the r-values for hip, knee, and ankle joint angles were 0.99, 0.99, and 0.96, and the r-values for joint moments were 0.92, 0.96, and 0.85. In the present study, there was no significant difference (*p* > 0.05) between the IMU system and the Mocap system in calculating lower limb joint angles and moments. (4) Conclusions: This study confirms the accuracy of the IMU in assessing lower limb muscle strength in the elderly. It provides a portable and accurate alternative for the assessment of lower limb muscle strength in the elderly.

## 1. Introduction

Older adults often face weakened lower extremity muscle strength due to aging, lifestyle changes, and underlying diseases [1,2]. This decline impacts basic movements like sitting and standing, which are crucial for independent living, and increases the risk of falls, injuries, and the need for long-term rehabilitation [3,4]. Studies indicate a strong link between lower limb muscle strength and fall risk [5], highlighting the need for accurate assessment and intervention. Traditional methods for assessing muscle strength, such as manual tests, are limited by operator skill and testing conditions, which can affect accuracy and reproducibility [6,7]. Therefore, new techniques are needed to improve assessment reliability [8]. Recent advancements include the use of machine learning and computer vision for real-time assessment of muscle strength during sit-to-stand transfers (STS). These technologies combine sensors and algorithms to provide objective data and track muscle strength changes over time, enabling more precise and individualized rehabilitation plans [9]. To enhance the quality of life of patients and maintain their independence, healthcare professionals should use comprehensive muscle strength assessments to create tailored exercise programs and daily activity recommendations, which can help slow muscle strength decline and reduce fall risk [10].

The STS test is a common, simple way to assess older adults’ functional capacity, but it lacks detailed quantitative data like force output, velocity, and acceleration [11]. This limits its effectiveness in accurately evaluating muscle strength and creating effective rehabilitation programs. Yoshioka et al. [12] found that while the STS test is useful, it does not allow for real-time monitoring of muscle strength changes, which hampers the development of personalized interventions. To overcome these limitations, researchers are turning to advanced technologies such as inertial measurement units (IMUs). IMUs, which include accelerometers, gyroscopes, and magnetometers, provide real-time kinematic data like acceleration and angular velocity. They are portable, cost-effective, and easy to use. Garcia [13] demonstrated their effectiveness in analyzing gait, and Li [14] showed their potential for assessing fall risk and motor function. Using IMUs in STS assessments can enhance accuracy by monitoring muscle force and kinematic changes in real time, aiding in the development of tailored rehabilitation plans and improving the quality of life for older adults [15]. This technology addresses limitations in current methods and offers new possibilities for muscle strength assessment and elderly health management.

Infrared motion capture technology is recognized for its accuracy in assessing lower limb muscle strength in the elderly but is limited by high costs, complex operation, and cumbersome testing processes [16]. In contrast, inertial measurement sensors offer a compact, portable, low-cost, and user-friendly alternative. These sensors monitor motor activity and provide real-time motion-related data [17]. This study aims to explore the feasibility of using inertial measurement sensors for assessing lower limb muscle strength during the sit-to-stand (STS) test, comparing them with infrared motion capture to validate their accuracy and reliability. The goal is to provide a practical and reliable option for daily health monitoring and clinical practice, potentially enhancing sports biomechanics research and health management. The sensors’ ability to capture dynamic parameters like attitude, acceleration, and angular velocity during STS tests could improve personalized rehabilitation programs and the overall quality of life for older adults.

## 2. Participants and Method

### 2.1. Participants

According to previous studies, using G-Power software (Version 3.1, Heinrich Heine University, Düsseldorf, Germany), correlation analysis was used to test the sample size first, and a minimum of 26 participants were needed to detect the expected differences in the validity and reliability of the IMU in healthy individuals with an effect size of 0.5, a test power of 0.8, and a test standard of 0.05 [18,19]. Twenty-eight subjects were finally included in this study. Their basic information is shown in Table 1.

The inclusion criteria of the subjects were as follows: Inclusion criteria: (1) age: 60–70 years old; (2) BMI ≤ 28; (3) no history of new lower extremity joint injury, fracture, or other neurological, musculoskeletal, or skeletal disease affecting motor function within the last six months (stroke, myocardial infarction, Parkinson’s disease, low back pain with a VAS score greater than 3, osteoarthritis of the knee, etc.). Exclusion criteria: (1) Simple Physical Performance Battery (SPPB) score < 7 or inability to perform STS test; (2) cognitive impairment (Measured Mental State Examination (MMSE) score < 27); (3) uncontrolled high blood pressure (> 200/110 mm Hg). The experimental protocol was approved by the Ethics Committee of Shanghai Sport University (No. 102772024RT064). Before the formal experiment, subjects were informed of the purpose of the experiment, the experimental protocol, the specific procedures of the experiment, and the precautions to be taken, and their consent was obtained and a subject informed consent form was signed.

### 2.2. Method

#### 2.2.1. Instrumentation

**Figure 1:**
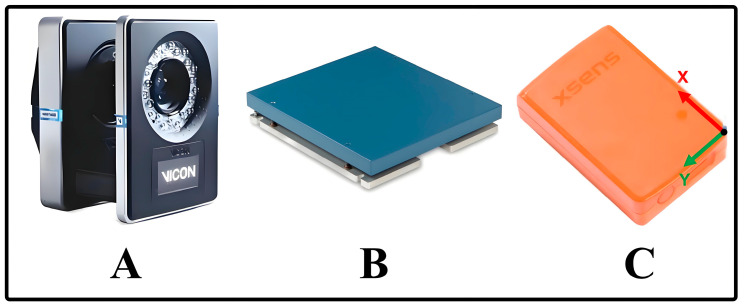
Experimental flow chart ((**A**): Vicon T40 motion capture; (**B**): AMTI OR6-7 force tables; (**C**): Movella Xsens MTw).

The experiment was conducted in the Physical Force Hall of Shanghai Sport University. A Vicon infrared motion capture system (Vicon T40, Oxford, UK) was used to collect the kinematic data of the three joints of the torso and the lower limbs, namely, the hip, knee, and ankle, during the STS test of the subjects at a sampling frequency of 200 Hz (see Figure 1A). Two 40 cm × 60 cm 3D force tables (AMTI OR6-7, Watertown, MA, USA) were used to synchronously collect the ground reaction force during a subject’s test at a sampling frequency of 1000 Hz (see Figure 1B). Torso, thigh, and calf kinematic data during the STS test were acquired using three Xsens MTw inertial measurement units (Movella, Enschede, The Netherlands) at a sampling frequency of 100 HZ (see Figure 1C). Each IMU included a triaxial accelerometer (range: ±160 m/s^2^), a triaxial gyroscope (range: ±2000°/s), and a triaxial magnetometer (range: ±1.9 Gauss).

#### 2.2.2. Test Protocols

**Figure 2:**
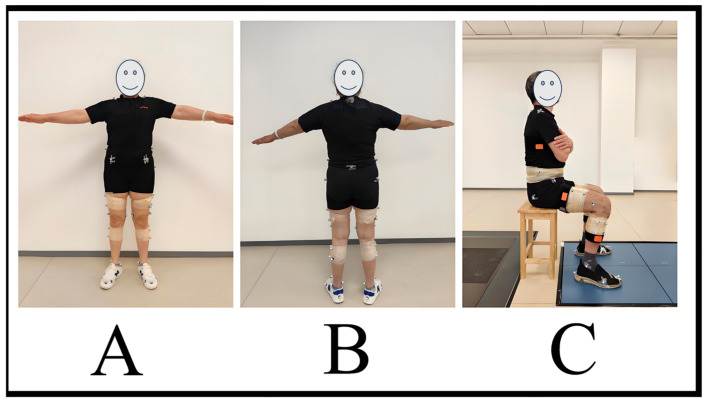
(**A**) Marker configuration front view. (**B**) Marker configuration back view. (**C**) Sensor configuration and test initial posture.

Subjects completed the informed consent form and other basic information and wore a uniform and tight-fitting shorts, and the experimenter measured the height and weight of the subjects using standard methods. Marker dots were pasted (14 mm in diameter), and IMUs were placed on the subjects by the designated experimenter, with a total of 42 marker dots in the standardized configurations (Figure 2A,B). Three IMUs were secured to the right side of the thigh, the middle of the lateral side of the calf, and the lateral side of the torso via a strap, so that the sensor’s long axis aligned with the long axis of the link (Figure 2C). Subsequently, the experimenter explained the test maneuvers, the subjects were familiarized with the test maneuvers 3 times, and the subjects walked around the laboratory for 5 min to warm up.

During the experiment, a Vicon infrared motion capture system was first used to capture the subject in a static calibrated pose for later modeling in the V3D software (Version 2023, C-Motion Inc., Germantown, MD, USA). The IMU was reset in this pose for alignment so that the 3 sensors had the same original pose. Subjects were asked to stand in a standard anatomical posture facing the front of a stool, with feet shoulder-width apart, arms open to 45°, and palms facing forward, and remain stationary.

The subject then sat on a 45 cm high stool with their torso perpendicular to the floor, feet on the force platform, and arms crossed in front of the chest. The STS test involves the subject performing the movement from sitting to standing as quickly as possible after hearing the command “start”. During this process, the Mocap system, including the force platform, and the IMU simultaneously collect kinematic and kinetic data from the subject during the STS test. The test was repeated 3 times with a 3 min rest period between each test.

#### 2.2.3. Data Collection and Processing

High-speed infrared motion capture and force table data: Vicon Nexus software (Version 2.16, Oxford Metrics, Oxford, UK) was used to fill in the points and intercept the raw data collected. The intercepted data were exported to C3D-format files and imported into Visual 3D software (Version 2023, C-Motion Inc., Germantown, MD, USA) to build a human body segment model. The human body model consists of eight segments, namely, the torso, pelvis, bilateral thighs, bilateral calves, and bilateral feet. The data were filtered using a Butterworth fourth-order low-pass filter with a kinematic cutoff frequency of 20 Hz and a force table cutoff frequency of 100 Hz [20], and the values of hip, knee, and ankle joint angles and triple joint moments were calculated using Visual 3D software. The hip angle was redefined in terms of the angle between the trunk and the femur to agree with the hip angle calculated by the sensors system. Moments were normalized by height and weight.

IMU data: The IMU provides real-time output of sensor fusion data, including Euler angles, quaternions, acceleration, and angular velocity. Since the STS process is mainly performed by the flexion and extension muscles of the lower limb joints, we only selected the kinematic data in the sagittal plane for this study. The sit–stand transfer phase was divided by the change in the Euler angle output from the IMU at the thigh in the sagittal plane: the maximum value of the IMU Euler angle corresponded to the sitting position, and the minimum value of the Euler angle corresponded to the end of the standing-up phase. Data analysis was performed using MATLAB, the joint flexion and extension angles, output by the IMU system, were calculated based on the quaternions output by the IMUs of adjacent links, and the joint angle data were interpolated and time-normalized. The lower limb joint moments after leaving the seat can be calculated by the Lagrange equation [21], which is a relatively simple method based on the concept of system energy, and parameters such as moments and ground reaction forces can be deduced by using only the link angles as inputs.

In this paper, we used the measured data of the infrared motion capture system as the gold standard and compared them to the estimated data of the IMU system to carry out the validity test of IMU-based devices for assessing lower limb muscle strength.

### 2.3. Statistical Analysis

Descriptive statistics of the obtained kinematic and kinetic data were expressed as mean ± standard deviation (Mean ± SD). The S-W (Shapiro–Wilk) test was used to determine the normality of the statistical data, and all data were normally distributed.

Data were statistically analyzed using Matlab (Version 2023b, MathWorks Inc., Natick, MA, USA) and SPSS 26.0 (IBM SPSS Inc., Chicago, IL, USA) software. In order to verify the validity of IMU sensors for lower limb muscle strength assessments in older adults, the agreement between the two systems for the estimation of peak moments at the hip, knee, and ankle joints in the STS test was analyzed using intraclass correlation coefficients (ICCs) and Bland–Altman plots, with ICCs of 0.00–0.39 indicating a weak agreement, 0.40–0.73 indicating a moderate agreement, 0.74–0.89 indicating a good agreement good, and 0.90–1.00 indicating excellent agreement [22]. The Spearman correlation coefficient (r) was used to analyze the correlation of peak moments between the two systems, where r-values of 0.00–0.49 indicate weak correlation, 0.50–0.74 indicate moderate correlation, and 0.75–1.00 indicate strong correlation [23]. Differences in peak joint angles and peak moments between the two systems were analyzed using the paired samples *t*-test and the root mean square error (RMSE) with a significance level of 0.05. The smaller the value of the RMSE was, the closer the measurements were between the two systems; and the larger the value of the RMSE was, the larger the difference in measurements between the two systems was.

## 3. Results

### 3.1. Peak Joint Angle Results within the Sagittal Plane of the Hip, Knee, and Ankle Joints Captured by the Two Systems

**Figure 3:**
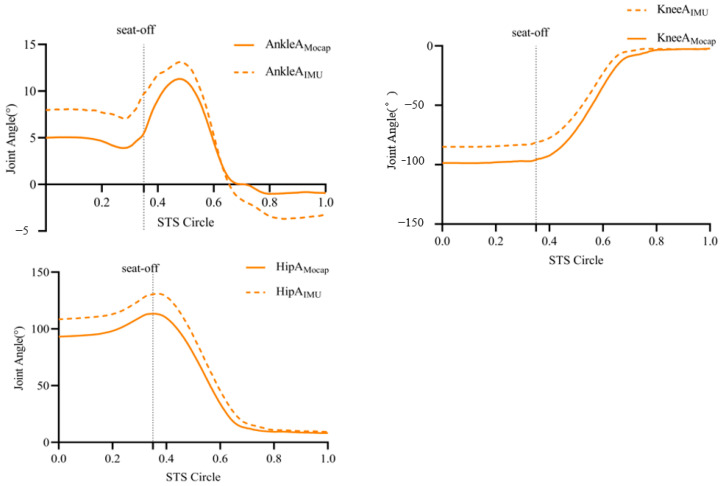
Ankle, knee, and hip joint angle changes in STS test.

The comparison of peak joint angles between the two systems, mocap and IMU, showed that there was no significant difference between the lower limb joint angles (*p* > 0.05), and the consistency of the IMU system’s estimation of lower limb hip and knee flexion–extension angles in the STS test of the elderly was excellent, with ICCs of >0.90, 0.990 (0.988, 0.992), and 0.989 (0.987, 0.991). The IMU system had a better agreement in estimating the flexion and extension angles of the ankle joint, with an ICC of 0.885 (0.862, 0.904). Compared with the motion capture system, the estimation of hip, knee, and ankle angles by the sensors reached strong correlation levels, with r-values > 0.75, 0.999, 0.989, and 0.885, respectively (Table 2, Figure 3).

### 3.2. Results of Peak Joint Moments in the Sagittal Plane of the Hip, Knee, and Ankle Joints Collected by the Two Systems

The paired samples *t*-test showed that there was no significant difference in the assessment of lower extremity hip, knee, and ankle flexion–extension moments during the STS test between the two systems in older adults (*p* > 0.05). The RMSEs were 0.16 N/kg, 0.22 N/kg, and 0.09 N/kg, respectively (Table 3).

#### 3.2.1. Consistency Test

**Figure 4:**
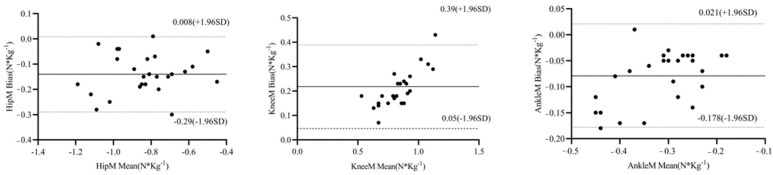
Bland–Altman analysis of peak moments between the two systems. Solid line: bias mean; dashed line: 95% CI.

Compared with the motion capture system, the IMU system had excellent agreement for the estimation of peak moments in the sagittal plane of the hip and knee joints, with ICCs of 0.94 (0.90, 0.98) and 0.92 (0.87, 0.97), respectively, and the estimation of ankle flexion–extension moments reached a higher level of accuracy, with an ICC of 0.89 (0.77, 0.95). In Table 3, the Bland–Altman analysis showed that almost all data were located within the bias mean ± 1.96 times the standard deviation, and the accuracy of the IMU for the estimation of hip–knee–ankle triple-joint moments was good (Table 3, Figure 4).

#### 3.2.2. Correlation Analysis

**Figure 5:**
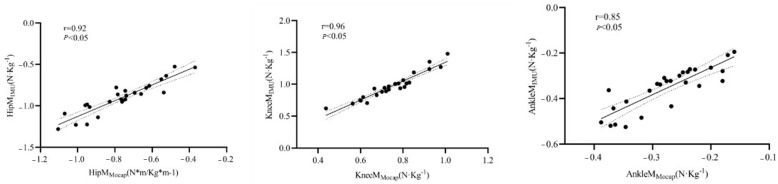
Pearson correlation analysis of the peak moments between the two systems, reporting the r-values and *p*-values. Solid line: regression line; dashed line: residual line.

Compared to the high-speed infrared motion capture system, the IMU system had extremely strong correlations for the estimation of peak moments in the sagittal plane of the hip, knee, and ankle joints, with r-values of > 0.75 for all joints, which were 0.92, 0.96, and 0.85, respectively (Table 3, Figure 5).

## 4. Discussion

This study compared the performance of the IMU system with the infrared motion capture (mocap) system in assessing lower limb muscle strength in older adults. The results showed that the IMU system did not differ significantly from the mocap system in measuring lower extremity joint angles during sit-to-stand transfers (STS). Specifically, the IMU system showed an ICC value of 0.885 in estimating the ankle joint angle, whereas it showed very high internal consistency in estimating the hip and knee joint angles, with ICC values exceeding 0.98. This indicates that the IMU system has good reliability and accuracy in capturing dynamic lower limb movements in older adults. In addition, the IMU system showed a strong correlation with the mocap system in the estimation of hip, knee, and ankle joint angles, which further validated its effectiveness in real-time monitoring of lower limb muscle performance in the elderly. These results provide a scientific basis for the widespread use of the IMU system as a novel and convenient measurement modality in clinical and daily health monitoring and also emphasize its importance in improving the quality of life and independence of older adults [24,25]. Traditional assessment methods such as manual strength testing suffer from a lack of accuracy and reproducibility [26]. In this study, we introduced an IMU system that was able to monitor the joint movements of older adults during STS in real time, providing important kinematic data. These data effectively captured the motion trajectories of the hip, knee, and ankle joints, analyzed the fluidity and stability of the motion, and provided a new perspective for assessing the quality of motion in older adults [27]. By comparing the angle measurements of the IMU with those of a conventional motion capture system, it is possible to identify trends in lower limb movement in older adults. Regular monitoring of changes in joint angles during STS allows for the early detection of signs of decreasing mobility so that targeted rehabilitation interventions, such as strength and balance training, can be implemented in a timely manner to improve the quality of life of older adults [28,29]. In addition, data analysis of the IMU system helps the healthcare team to develop a personalized rehabilitation plan to identify motor skill deficits and muscle imbalances so that targeted rehabilitation programs can be designed to enhance the stability and function of specific joints [30]. This study demonstrates that the application of IMU systems is not limited to clinical research but also has important social and economic implications. Its convenience and efficiency can significantly reduce the cost and time of data collection, promote the innovation of health management and rehabilitation strategies for the elderly, and provide more effective services for society [31].

We also found that the correlation between the IMU system and the motion capture system was extremely strong in assessing the joint motion moment, with correlation coefficients of 0.92, 0.96, and 0.85 for the hip, knee, and ankle joints, respectively, which suggests that the IMU system is able to accurately reflect the trend in joint motion. These results emphasize the importance of moments in assessing the function of the lower limbs in the elderly, which can monitor the functional changes in lower limb activities in real time and provide an important basis for the development of personalized rehabilitation training and health management plans [32,33]. By monitoring motor moments through the IMU system, we can effectively improve the daily mobility of older adults and reduce the risk of functional decline, thus enhancing their quality of life [34]. Previous studies have highlighted the strong link between joint moments and muscle strength, primarily using traditional motion capture systems. Our research validates the consistency and comparability of an IMU-based system for measuring joint moments. Our data analysis and Bland–Altman plots demonstrate that IMUs provide stable and accurate measurements with high internal consistency and repeatability. This system’s real-time monitoring capabilities are particularly beneficial for older adults, allowing for timely adjustments to rehabilitation programs [35]. Additionally, IMUs are easy to use and deploy, making them suitable for community healthcare and long-term care settings [36]. In practice, the IMU’s performance can be affected by factors such as sensor drift and environmental noise, so calibration and filtering techniques need to be used to ensure the accuracy of the measurements. This study uses the Movella Xsens MTw sensor, which uses a Kalman filter to solve the posture of the limb link relative to space with good accuracy [37], which is beneficial for the subsequent calculation of the joint angles; at the same time, it can be synchronized with the VICON motion capture system, which makes it technically possible to achieve a synchronized analysis of the human body’s motion. In addition, the sliding of the IMU with respect to the clothes and the vibration of the IMU itself during the movement can negatively affect the measurement results, which we minimized in our experiments by asking participants to wear standard experimental shorts and firmly fixing the IMU in a fixed position on the limb link.

Therefore, kinetic joint moment assessment systems based on inertial measurement sensors provide a new and efficient option for assessing lower limb muscle strength in the elderly. The subjects in this paper were 60–70 years of age, and in the future, older subjects will be considered for inclusion in the study to increase the applicability of the IMU for lower extremity muscle strength estimation in the elderly. Our study not only emphasizes its theoretical importance but also empirically demonstrates its feasibility and superiority in practical applications, providing a solid foundation for further promotion and improvement in the future.

## 5. Conclusions

This study validated the effectiveness and reliability of an IMU-based measurement method for assessing lower limb muscle strength in older adults. The IMU system demonstrated a comparable performance to high-speed infrared motion capture, with strong consistency in joint angle and moment measurements at the hip, knee, and ankle. It accurately captured joint dynamics during the STS test, providing stable measurements that are essential for personalized rehabilitation and monitoring muscle strength. The IMU’s ease of use and real-time monitoring offer significant advantages for clinical and daily health management, reducing data collection costs and time. This technology not only enhances assessment accuracy but also supports sustainable health management and rehabilitation strategies for the elderly.

## Figures and Tables

**Table 1:** Basic information on subjects.

Number of Persons (Male/Female)	Age (Years)	Height (m)	Weight (kg)	BMI (kg/m^2^)	SPPB (Score)
28 (13/15)	64.79 ± 4.58	1.61 ± 0.07	63.15 ± 7.69	24.3 ± 1.96	10.6 ± 0.9

**Table 2:** Peak joint angles for both systems.

Joint	P-A_mocap_ (°)	P-A_IMU_ (°)	RMSE (°)	*p*-Value	r	ICC (95%CI)
Ankle	10.67 ± 2.98	13.02 ± 2.84	2.5	0.091	0.96 *	0.885 (0.862, 0.904)
Knee	−99.2 ± 15.37	−85.95 ± 18.67	10.2	0.125	0.99 *	0.989 (0.987, 0.991)
Hip	113.3 ± 19.18	130.4 ± 19.60	12.4	0.372	0.99 *	0.990 (0.988, 0.992)

P-A_mocap_—peak angle by motion capture system; P-A_IMU_—peak angle by IMU system; RMSE—rms error; *p*-Value—*c*orresponding to paired *t*-tests; r—Spearman’s correlation coefficient; * represents *p* < 0.05; ICC and 95%CI; ICC—intraclass correlation coefficient with extreme 95% confidence intervals.

**Table 3:** Peak joint moments for both systems.

Joint	P-M_mocap_ (N/kg)	P-M_IMU_ (N/kg)	RMSE (N/kg)	*p*-Value	r	ICC (95%CI)
Ankle	−0.27 ±0.12	−0.31 ± 0.09	0.09	0.08	0.85 *	0.89 (0.77, 0.95)
Knee	0.83 ± 0.13	0.96 ± 0.21	0.22	0.68	0.96 *	0.92 (0.87, 0.97)
Hip	−0.86 ± 0.18	−0.90 ± 0.19	0.16	0.92	0.92 *	0.94 (0.90, 0.98)

P-A_mocap_—peak angle by motion capture system; P-A_IMU_—peak angle by IMU system; RMSE—rms error; *p*-Value—corresponding to paired *t*-tests; r—Spearman’s correlation coefficient; * represents *p* < 0.05; ICC and 95%; ICC—intracluster correlation coefficient with extreme 95% confidence intervals.

## Data Availability

The data that support the findings of this study are available on request from the corresponding author.
